# Lead and Cadmium Levels and Balance and Vestibular Dysfunction among Adult Participants in the National Health and Nutrition Examination Survey (NHANES) 1999–2004

**DOI:** 10.1289/ehp.1103643

**Published:** 2012-01-03

**Authors:** Kyoung-Bok Min, Kyung-Jong Lee, Jae-Beom Park, Jin-Young Min

**Affiliations:** 1 Department of Occupational and Environmental Medicine, Ajou University School of Medicine, Suwon, Republic of Korea; 2 Institute of Health and Environment, Seoul National University, Seoul, Republic of Korea

**Keywords:** cadmium, lead, ototoxicity, neurotoxicity, vestibular disturbance

## Abstract

Background: Few studies have been conducted to identify risk factors for balance and vestibular dysfunction in general populations, but previous studies have reported evidence of adverse effects of lead and cadmium on balance control in high-risk groups.

Objective: We evaluated the relationship between blood lead and cadmium levels and balance and vestibular dysfunction in a general population study.

Methods: We analyzed data from the 1999–2004 National Health and Nutrition Examination Survey (NHANES) of 5,574 adults ≥ 40 years of age. Balance dysfunction was evaluated by the Romberg Test of Standing Balance on Firm and Compliant Support Surfaces, which examines the ability to stand unassisted using four test conditions to evaluate vestibular system, vision, and proprioception inputs that contribute to balance. Blood levels of lead and cadmium were measured by atomic absorption spectrometry. Associations were estimated using logistic regression models adjusted for potential confounders. Associations with time to loss of balance were estimated using adjusted Cox proportional hazard models.

Results: The adjusted odds ratio (OR) for balance dysfunction in association with the highest quintile (3.3–48 µg/dL) versus the lowest quintile (< 1.2 µg/dL) of lead was 1.42 [95% confidence interval (CI): 1.07, 1.89]. The corresponding OR for cadmium (0.9–7.4 µg/L vs. < 0.2 µg/L) was 1.27 (95% CI: 1.01, 1.60). The adjusted hazard ratio for time to failure for the most physiologically challenging balance test among subjects with the highest vs. lowest quintiles of blood lead was 1.24 (95% CI: 1.04, 1.48). Cadmium levels were not associated with time to failure.

Conclusions: Our findings suggest that blood lead and cadmium levels may be associated with balance and vestibular dysfunction in a general sample of U.S. adults.

Many studies have examined the effects of lead and cadmium exposures on neurobehavioral functions, such as declines in cognitive ability and visuomotor performance (Balbus-Kornfeld et al. 1995; Viaene et al. 2000), but there is increasing evidence suggesting that exposures are associated with hearing loss and alterations in auditory evoked potentials (Araki et al. 1986; Forst et al. 1997; Murata et al. 1993; Ozcaglar et al. 2001). In addition, several studies have reported that workers with occupational exposure to lead have significantly poorer postural balance responses than do unexposed workers (Chia et al. 1994, 1996; Dick et al. 1999; Iwata et al. 2005; Linz et al. 1992; Ratzon et al. 2000; Yokoyama et al. 1997, 2002). Impaired postural stability also has been associated with blood lead concentrations in children (Bhattacharya et al. 1990, 1995, 2006, 2007; Després et al. 2005). However, relatively few studies have evaluated associations between cadmium exposure and balance disturbance (Kilburn and McKinley 1996; Viaene et al. 2000).

An individual’s ability to maintain balance is an essential component for nearly all daily life activities. The control of balance depends on sensory inputs from the vestibular and visual systems, neural processing centers throughout the central nervous system, and motor outputs (the proprioceptive system). Functional declines in any of these systems can lead to balance deficits (Horak 2006; Sturnieks et al. 2008). Although the prevalence of balance dysfunction in the United States is unknown, data from the National Health and Nutrition Examination Survey (NHANES) suggest that 35% of U.S. adults ≥ 40 years of age (69 million people) show objective evidence of vestibular dysfunction (Agrawal et al. 2009).

Few studies of risk factors for decreased balance andvestibular function have been conducted in the general population, despite the public health significance of these outcomes (Neuhauser 2007; Neuhauser et al. 2005). Furthermore, it is unclear whether exposures to lead and cadmium affect balance or vestibular function in the general population. Therefore, we investigated the relationship between blood lead and cadmium levels and balance or vestibular dysfunction (identified based on a modified Romberg Test for Standing Balance on Firm and Compliant Support Surfaces) among adults who participated in NHANES 1999–2004.

## Methods and Materials

*Study population.* The NHANES, conducted by the Centers for Disease Control and Prevention (CDC), is a nationally representative survey of the noninstitutionalized civilian population in the United States. The 1999–2004 NHANES study protocols were approved by the National Center for Health Statistics Institutional Review Board. All participants gave oral and written informed consent.

The present study used data on balance and blood levels of lead and cadmium that were measured concurrently in NHANES participants (1999–2004). The NHANES Balance Component was assessed in a 50% sample of U.S. adults 40–69 years of age in the 1999–2000 survey (*n* = 851), and in all adult participants ≥ 40 years of age in the NHANES 2001–2002 (*n* = 2,541) and the 2003–2004 (*n* = 2,459) surveys. Of the 5,851 total participants, 5,574 (95.3% of those who were eligible) also had blood lead and cadmium measurements and data for all covariate variables.

*Balance examination.* For safety reasons, particpants were excluded from the balance test if, at the time of the exam, they felt unable to stand on their own, had current symptoms of dizziness or lightheadedness, weighed more than 275 lb, had a waist circumference that did not accommodate any standard-sized safety gait belt, needed a leg brace to stand unassisted, or had had a foot or leg amputation. Those who were blind or who were sufficiently visually impaired to require assistance in locating the exam room were also excluded.

The Romberg Test of Standing Balance on Firm and Compliant Support Surfaces (CDC 2001b) measures the participant’s ability to maintain balance under four test conditions, which are ordered in increasing levels of difficulty. In brief, in test condition 1, subjects must maintain their balance while standing (with their feet together and arms folded across the waist, holding each elbow with the opposite hand) for 15 sec. In test condition 2, subjects must maintain their balance while standing for 15 sec with their eyes closed so that only vestibular and proprioceptive (i.e., leg muscle position sense) information is available. In test condition 3, subjects must maintain their balance while standing for 30 sec on a foam-padded surface, which reduces proprioceptive input but does not affect visual or vestibular input. In test condition 4, subjects must maintain their balance while standing for 30 sec on a foam-padded surface with their eyes closed, so that input is available from the vestibular system only.

Each condition is scored on a pass or fail basis; failure was defined as movement of the feet from the initial test position; movement of the arms off the waist; opening the eyes during test conditions 2 or 4; or any intervention by the health technologist to stop the subject from falling within the maximum time period for the individual test. Subjects who fail a test condition are given one opportunity to repeat the test. Because each successive test condition from 1 to 4 is progressively more difficult than the condition preceding it, the balance-testing component is ended whenever a subject fails to pass a condition. The time to failure (i.e., loss of balance) is also recorded for test condition 4, with those who passed the test assigned the maximum value of 30 sec.

*Measurement of blood lead and cadmium levels.* Whole blood specimens were stored frozen (–20°C) until they were shipped to the National Center for Environmental Health for testing. Blood lead and cadmium levels were measured using a multielement atomic absorption spectrometer with Zeeman background correction (SIMAA 6000 model; Perkin-Elmer, Norwalk, CT, USA). The analytical laboratory (Division of Laboratory Sciences, National Center for Environmental Health, Atlanta, GA, USA) followed extensive quality control procedures.

For external calibration of blood lead and cadmium levels (CDC 2001a, 2004), standard reference materials from the National Institute of Standards and Technology (NIST; Gaithersburg, MD, USA) were used. The limit of detection (LOD) for blood lead was 0.3 µg/dL in NHANES (1999–2004). For blood cadmium, the LOD was 0.3 µg/L in NHANES (1999–2002) and 0.2 µg/L in NHANES (2003–2004). For participants with blood cadmium or lead levels below the LOD, a level equal to the LOD divided by the square root of 2 was used (CDC 2005; Hornung and Reed 1990). Interassay coefficients of variation ranged from 3.1% to 4.0% for lead and from 3.2% to 9.4% for cadmium.

*Other variables of interests.* We also evaluated other factors that might be associated with balance dysfunction and exposure to lead or cadmium, including age (40–59 years, 60–79 years, and ≥ 80 years), sex (male and female), race/ethnicity (non-Hispanic white, non-Hispanic black, Mexican American, and other), and education (< high school, high school diploma, and > high school). Smoking and consumption of alcohol were modeled as pack-years and ounces per day, respectively (continuous variables, those who never smoked or who never used alcohol were assigned values of 0). History of stroke was defined based on a self-reported physician’s diagnosis. History of diabetes mellitus was defined based on a self-reported physician’s diagnosis or an 8-hr fasting serum glucose level ≥ 126 mg/dL at the time of the examination. Dietary calcium intake was categorized as ≥ 1,000 mg or < 1,000 mg, and iron intake was categorized as ≥ 8 mg or < 8 mg.

*Statistical analysis.* To account for the complex sampling design, weighted estimates of the population parameters were computed. Because of differences in the sampling frames for the balance examination, we combined the 1999–2000 and 2001–2002 balance data sets using special 4-year balance subsample weights; 6-year data analyses (1999–2004) were performed by combining the 4-year balance subsample weights with the 2003–2004 examination weights according to NHANES analytic guidelines (CDC 2006). We performed all analyses using SAS 9.2 (SAS Institute Inc., Cary, NC, USA) and set statistical significance at α = 0.05. All statistical analyses were 2-sided tests.

Statistical differences in demographic characteristics between participants with and without balance dysfunction were evaluated. Because of their right-skewed distribution, blood concentrations of lead and cadmium were log transformed and were categorized into quintiles based on their weighted distributions.

Binary logistic regression using PROC SURVEYLOGISTIC procedures (SAS Institute Inc.) was conducted to compare the prevalence of balance dysfunction for each quintile of lead or cadmium distribution with the lowest quintile of exposure. We used the Hosmer–Lemeshow goodness-of-fit test (Hosmer and Lemeshow 2000), Pearson residual normality plots, and their corresponding histograms to assess model fit. To estimate odds ratios (ORs) for failure of each individual test condition, we conducted multinomial logit regression analyses using the generalized logit function (GLOGIT) of PROC SURVEYLOGISTIC (SAS Institute Inc.). Here, because we did not have sufficient power to estimate associations between quintiles of exposure and case 1, given that only seven participants failed, estimates for case 1 from the results were excluded. We calculated adjusted ORs and their 95% confidence intervals (CIs) for each logistic regression. The Taylor series linearization method (Rust 1985) was used for complex sample variance estimation.

We estimated associations between failure time for balance test condition 4 and blood lead and cadmium levels using Cox proportional hazards models (Cox 1972), with outcomes censored at 30 sec for participants who completed the test without losing their balance. We checked the proportional hazards assumption using log-negative–log survival curves, which were approximately parallel, indicating that the proportional hazards assumption was not violated. The Cox proportional hazards model was performed by PROC SURVEYPHREG procedures (SAS Institute Inc.).

Statistical models were adjusted for age, sex, race/ethnicity, education, pack-years of smoking, alcohol consumption (ounces per day), histories of stroke and diabetes, and intakes of calcium and iron.

## Results

Of the 5,574 participants, 3,084 successfully completed all four balance test conditions and were designated as without balance dysfunction, whereas 2,490 failed to pass one of the test conditions, including 7 who failed to pass test condition 1 (case 1); 163 who passed condition 1 but failed to pass test condition 2 (case 2); 86 who passed conditions 1 and 2 but failed to pass test condition 3 (case 3); and 2,234 who passed conditions 1, 2, and 3 but failed to pass test condition 4 (case 4) (Table 1). Compared with participants who displayed no balance dysfunction, those with a balance dysfunction were older (54 years vs. 65 years). Participants with less education, the presence of diabetes and stroke, and low calcium and iron intakes were more likely to display balance dysfunction. There were no statistically significant differences in sex, race/ethnicity, smoking pack-years, or average daily alcohol consumption between the two groups. The means (95% CI) of the blood lead and cadmium levels were 2.21 µg/dL (95% CI: 2.13, 2.28) and 0.61 µg/L (95% CI: 0.58, 0.64), respectively. Participants with versus those without vestibular dysfunction had higher mean blood lead (2.39 µg/dL, 95% CI: 2.29, 2.49 vs. 2.09 µg/dL, 95% CI: 2.01, 2.18) and cadmium (0.64 µg/L, 95% CI: 0.61, 0.67 vs. 0.59 µg/L, 95% CI: 0.54, 0.63) levels.

**Table 1:** Characteristics of the NHANES 1999–2004 study population*^a^* based on the presence or absence of balance dysfunction.

Without balance dysfunction (*n* = 3,084)	With balance dysfunction*b*								
	Characteristic	Case 1 (*n* = 7)	Case 2 (*n* = 163)	Case 3 (*n* = 86)	Case 4 (*n* = 2,234)	Total (*n* = 2,490)
Age (years)												
40–59		2,064 (66.93)		—		19 (11.66)		6 (6.98)		708 (31.69)		733 (50.18)
60–79		956 (31.00)		3 (42.86)		82 (50.31)		36 (41.86)		1,184 (53.00)		1,305 (40.56)
≥ 80		64 (2.08)		4 (57.14)		62 (38.04)		44 (51.16)		342 (15.31)		452 (9.26)
Sex												
Male		1,578 (51.17)		3 (42.86)		94 (57.67)		37 (43.02)		1,104 (49.42)		1,238 (50.52)
Female		1,506 (48.83)		4 (57.14)		69 (42.33)		49 (56.98)		1,130 (50.58)		1,252 (49.48)
Race/ethnicity												
White		1,687 (54.70)		3 (42.86)		109 (66.87)		56 (65.12)		1,270 (56.85)		1,438 (56.06)
Black		588 (19.07)		3 (42.86)		15 (9.20)		15 (17.44)		355 (15.89)		388 (17.51)
Hispanic		722 (23.41)		1 (14.29)		39 (23.93)		14 (16.28)		544 (24.35)		598 (23.68)
Others		87 (2.82)		—		—		1 (1.16)		65 (2.91)		66 (2.74)
Education												
< High school		770 (24.97)		5 (71.43)		70 (42.94)		41 (47.67)		823 (36.84)		939 (30.66)
High school		710 (23.02)		2 (28.57)		41 (25.15)		17 (19.77)		569 (25.47)		629 (24.02)
≥ College		1,604 (52.01)		—		52 (31.90)		28 (32.56)		842 (37.69)		922 (45.32)
History of diabetes												
Yes		334 (10.83)		1 (14.29)		49 (30.06)		26 (30.23)		435 (19.47)		511 (15.16)
No		2,750 (89.17)		6 (85.71)		114 (69.94)		60 (69.77)		1,799 (80.53)		1,979 (84.84)
History of stroke												
Yes		59 (1.91)		—		17 (10.43)		14 (16.28)		123 (5.51)		154 (3.82)
No		3,025 (98.09)		7 (100.00)		146 (89.57)		72 (83.72)		2,111 (94.49)		2,336 (96.18)
Calcium intake (mg)												
≥ 1,000		787 (25.52)		2 (28.57)		31 (19.02)		8 (9.30)		481 (21.53)		522 (23.48)
< 1,000		2,297 (74.48)		5 (71.43)		132 (80.98)		78 (90.70)		1,753 (78.47)		1,968 (76.52)
Iron intake (mg)												
≥ 8		2,561 (83.04)		7 (100.00)		125 (76.69)		60 (69.77)		1,789 (80.08)		1,981 (81.49)
< 8		523 (16.96)		—		38 (23.31)		26 (30.23)		445 (19.92)		509 (18.51)
Smoking (pack-years)		3.40 (2.79, 4.00)		6.0 (0.00, 17.06)		3.64 (0.16, 7.11)		1.64 (0.00, 3.87)		3.82 (3.17, 4.46)		3.76 (3.07, 4.45)
Alcohol (ounce/day)		0.44 (0.37, 0.50)		—		0.38 (–0.07, 0.82)		0.17 (0.07, 0.28)		0.39 (0.33, 0.46)		0.39 (0.32, 0.46)
Blood lead (µg/dL)		2.09 (2.01, 2.18)		4.64 (2.61, 6.67)		2.46 (2,13, 2.79)		2.78 (2.31, 3.25)		2.38 (2.27, 2.48)		2.39 (2.29, 2.49)
Blood cadmium (µg/L)		0.59 (0.54, 0.63)		0.96 (0.29, 1.63)		0.67 (0.49, 0.84)		0.62 (0.48, 0.75)		0.63 (0.61, 0.66)		0.64 (0.61, 0.67)
—, no subjects. **a**For categorical variables, values are expressed as the number of participants (percentage); for continuous variables, values are the weighted mean (95% CIs). **b**Includes subjects who failed to complete at least one test; case 1 includes subjects who failed to pass test condition 1; case 2 includes subjects who passed test condition 1 but failed to pass test condition 2; case 3 includes subjects who passed test conditions 1 and 2 but failed to pass test condition 3; case 4 includes subjects who passed test conditions 1, 2, and 3 but failed to pass test condition 4.												

Fully adjusted ORs for balance dysfunction, comparing blood lead quintiles 2, 3, 4, and 5 with the lowest quintile, were 1.14 (95% CI: 0.94, 1.38), 0.96 (95% CI: 0.74, 1.24), 1.18 (95% CI: 0.95, 1.47), and 1.42 (95% CI: 1.07, 1.89), respectively, (Table 2). Corresponding ORs for blood cadmium were 1.27 (95% CI: 1.03, 1.56), 0.97 (95% CI: 0.83, 1.15), 1.28 (95% CI: 1.01, 1.61), and 1.27 (95% CI: 1.01, 1.60), respectively. Pearson residual normality plots and corresponding histograms, as well as Hosmer and Lemeshow goodness-of-fit tests (*p* = 0.69 for lead and *p* = 0.47 for cadmium) indicated that models fit reasonably well (data not shown).

**Table 2:** ORs*^a^* of balance dysfunction by quintile of blood lead and cadmium levels.

Quintile	With/without balance dysfunction	OR (95% CIs)
Lead (µg/dL)				
1 (< 1.2)		440 696		1 (Reference)
2 (1.3–1.7)		501 684		1.14 (0.94, 1.38)
3 (1.8–2.2)		414 561		0.96 (0.74, 1.24)
4 (2.3–3.2)		542 607		1.18 (0.95, 1.47)
5 (3.3–48)		593 536		1.42 (1.07, 1.89)
Cadmium (µg/L)				
1 (< 0.2)		440 696		1 (Reference)
2 (0.3–0.3)		501 684		1.27 (1.03, 1.56)
3 (0.4–0.5)		414 561		0.97 (0.83, 1.15)
4 (0.6–0.8)		542 607		1.28 (1.01, 1.61)
5 (0.9–7.4)		593 536		1.27 (1.01, 1.60)
**a**OR is the ratio of the odds of balance dysfunction for total participants who failed to complete at least one test (i.e., those with balance dysfunction, including all participants of cases 1, 2, 3, and 4, to the odds of balance dysfunction for those who passed all four stages of the balance test). The model was adjusted for age, sex, race/ethnicity, education, history of diabetes, history of stroke, smoking, alcohol consumption, calcium intake, and iron intake.				

Fully adjusted ORs for failure of each balance test comparing the highest with the lowest quintiles of blood lead concentrations were 1.38 (95% CI: 0.64, 2.94) for case 2, 2.17 (95% CI: 1.03, 4.59) for case 3, and 1.42 (95% CI: 1.07, 1.87) for case 4 (Table 3). The corresponding ORs for cadmium were 1.22 (95% CI: 0.55, 2.70) for case 2, 1.83 (95% CI: 0.61, 5.49) for case 3, and 1.27 (95% CI: 1.01, 1.61) for case 4.

**Table 3:** ORs of the failure or success of each test of balance dysfunction on increasing quintiles of blood lead and cadmium levels.

Case 2 (*n* = 163)			Case 3 (*n* = 86)			Case 4 (*n *= 2,234)		
Quintile	No. of subjects	OR (95% CIs)	No. of subjects	OR (95% CIs)	No. of subjects	OR (95% CIs)
Lead (µg/dL)												
1 (< 0.2)		27		Reference		13		Reference		400		Reference
2 (1.3–1.7)		32		1.68 (0.84, 3.38)		16		1.64 (0.74, 3.62)		452		1.11 (0.93, 1.34)
3 (1.8–2.2)		21		0.48 (0.20, 1.19)		12		1.09 (0.53, 2.27)		379		0.97 (0.76, 1.26)
4 (2.3–3.2)		35		0.95 (0.45, 2.05)		22		2.15 (1.01, 4.60)		485		1.19 (0.96, 1.47)
5 (3.3–48)		48		1.38 (0.64, 2.94)		23		2.17 (1.03, 4.59)		518		1.42 (1.07, 1.87)
Cadmium (µg/L)												
1 (< 0.2)		24		Reference		6		Reference		323		Reference
2 (0.3–0.3)		19		1.39 (0.81, 2.38)		17		4.33 (1.35, 13.83)		300		1.24 (0.99, 1.55)
3 (0.4–0.5)		62		1.33 (0.70, 2.53)		24		1.45 (0.56, 3.74)		716		0.96 (0.81, 1.13)
4 (0.6–0.8)		30		0.83 (0.39, 1.76)		21		3.01 (1.24, 7.33)		451		1.29 (1.01, 1.63)
5 (0.9–7.4)		28		1.22 (0.55, 2.70)		18		1.83 (0.61, 5.49)		444		1.27 (1.01, 1.61)
Number of subjects includes those participants with balance dysfunction who failed to complete at least one test; case 2 includes subjects who passed test condition 1 but failed to pass test condition 2; case 3 includes subjects who passed test conditions 1 and 2 but failed to pass test condition 3; case 4 includes subjects who passed test conditions 1, 2, and 3 but failed to pass test condition 4. The model was adjusted for age, sex, race/ethnicity, education, history of diabetes, history of stroke, smoking, alcohol consumption, calcium intake, and iron intake. No. subjects: number of subjects with balance dysfunction.												

Time to failure for balance test 4 was significantly higher among participants with the highest versus lowest quintile of lead exposure (hazard ratio = 1.24, 95% CI: 1.04, 1.48), but cadmium levels were not significantly associated with time to failure (Table 4).

**Table 4:** Hazard ratio (HR) of failure time of the balance test 4 associated with blood lead and cadmium levels.

Quintile	HR (95% CIs)	
Lead (µg/dL)		
1 (< 1.2)	Reference	
2 (1.3–1.7)	1.08	(0.94, 1.24)
3 (1.8–2.2)	0.99	(0.86, 1.15)
4 (2.3–3.2)	1.11	(0.96, 1.30)
5 (3.3–48)	1.24	(1.04, 1.48)
Cadmium (µg/L)		
1 (< 0.2)	Reference	
2 (0.3–0.3)	1.03	(0.91, 1.17)
3 (0.4–0.5)	0.94	(0.82, 1.08)
4 (0.6–0.8)	1.14	(0.97, 1.33)
5 (0.9–7.4)	1.09	(0.94, 1.27)
Participants who passed the test were censored at 39 sec. The model was adjusted for age, sex, race/ethnicity, education, history of diabetes, history of stroke, smoking, alcohol consumption, calcium intake, and iron intake.		

## Discussion

After adjusting for potential covariates, the prevalence of balance dysfunction was significantly higher among participants in the highest versus lowest quintiles of lead and cadmium (OR = 1.42, 95% CI: 1.07, 1.89 and OR = 1.27, 95% CI: 1.01, 1.60, respectively). However, lower quintiles of lead were not clearly associated with the outcome, and associations with cadmium were comparable for quintiles 2, 4, and 5, but absent for quintile 3. The hazard ratio for time to failure for balance test 4 was significantly higher among participants in the highest versus lowest quintiles of blood lead (hazard ratio = 1.24, 95% CI: 1.04, 1.48) only, while cadmium levels were not clearly associated with time to failure for test condition 4.

There is substantial evidence that implicates associations of impaired balance (Bhattacharya et al. 1990, 1995, 2006, 2007; Chia et al. 1994, 1996; Després et al. 2005; Dick et al. 1999; Iwata et al. 2005; Kilburn and McKinley 1996; Linz et al. 1992; Ratzon et al. 2000; Viaene et al. 2000; Yokoyama et al. 1997, 2002), hearing loss (Agirdir et al. 2002; Bertoni and Sprenkle 1988; Bleecker et al. 2003; de Abreu and Suzuki 2002; Forst et al. 1997; Hwang et al. 2009; Lasky et al. 2001; Ozcaglar et al. 2001; Yokoyama et al. 2002), and alterations in auditory evoked potential (Araki et al. 1986; Bleecker et al. 2003; Murata et al. 1993) with exposure to lead and cadmium. However, it is not known whether exposure to lead and cadmium induces vestibular dysfunction specifically.

To our knowledge, we are the first to demonstrate associations between impaired vestibular function and exposure to lead and cadmium in the general population. However, the clinical relevance of the outcomes we assessed is uncertain and it is not clear whether the balance test used reflects a defect in vestibular function specifically. Because postural balance is controlled by a complex interaction of multiple sensorimotor processes (i.e., the visual system, proprioception, and the vestibular system), balance, even with the limited input, can be maintained with the remaining afferents (Horak 2006). For example, test condition 4 was conducted on a foam-padded surface to reduce proprioception information; proprioception is still present but may be inaccurate, and the importance of the proprioceptive system to balance increases when visual information is also removed (Sahlstrand et al. 1978).

Our study has several limitations. First, because of its cross-sectional design, we can not confirm the temporal sequence of exposures and outcomes. However, it is unlikely that balance and vestibular dysfunction increases an individual’s exposure to lead and cadmium. Second, blood lead and cadmium levels alone are not an accurate reflection of lifetime exposures. Although blood measurements of both metals are commonly used in studies, and some studies have suggested that the effects of lead on postural stability or vestibular function are related more to recent increases in blood lead concentrations among exposed workers rather than to cumulative body burdens (Chia et al. 1996; Yokoyama et al. 1997), studies using markers that reflect cumulative exposure are needed. Third, we defined vestibular dysfunction based on the Romberg Test of Standing Balance, which is not a sensitive tool for diagnosing clinically relevant vestibular dysfunction. However, the test is commonly used as a standardized screening instrument in balance assessments in both clinical and research settings, and test results are considered when diagnosing vestibular dysfunction in clinical settings (Dobie 1997). Fourth, we could not control for exposure to cadmium and lead in the same model because of multicollinearity, and therefore could not estimate independent effects of lead and cadmium on balance/vestibular function. Finally, misclassification of self-reported data or possible bias due to uncontrolled confounding by unmeasured factors, such as family history and urbanization, cannot be ruled out.

In conclusion, we provide preliminary evidence of associations between blood levels of lead and cadmium and balance and vestibular dysfunction in a representative sample of U.S. adults ≥ 40 years of age. Although our findings require confirmation, they suggest that background exposures to lead and cadmium may be deleterious to the maintenance of balance and may be involved in the pathogenesis of vestibular dysfunction in the general population.
